# Combined measurement of soluble and cellular ICAM-1 among children with *Plasmodium falciparum *malaria in Uganda

**DOI:** 10.1186/1475-2875-9-233

**Published:** 2010-08-16

**Authors:** Christine M Cserti-Gazdewich, Walter H Dzik, Laura Erdman, Isaac Ssewanyana, Aggrey Dhabangi, Charles Musoke, Kevin C Kain

**Affiliations:** 1 Department of Laboratory Medicine & Pathobiology, University Health Network/University of Toronto, Toronto, Canada; 2 Department of Pathology, Massachusetts General Hospital, Boston, MA, USA; 3 McLaughlin-Rotman Centre for Global Health, University Health Network/University of Toronto, Toronto, Canada; 4 Joint Clinical Research Centre, Kampala, Uganda; 5 Makerere University, Kampala Uganda

## Abstract

**Background:**

Intercellular adhesion molecule-1 (ICAM-1) is a cytoadhesion molecule implicated in the pathogenesis of *Plasmodium falciparum *malaria. Elevated levels of soluble ICAM-1 (sICAM-1) have previously been reported with increased malaria disease severity. However, studies have not yet examined both sICAM-1 concentrations and monocyte ICAM-1 expression in the same cohort of patients. To better understand the relationship of soluble and cellular ICAM-1 measurements in malaria, both monocyte ICAM-1 expression and sICAM-1 concentration were measured in children with *P. falciparum *infection exhibiting a spectrum of clinical severity.

**Methods:**

Samples were analysed from 160 children, aged 0.5 to 10.8 years, with documented *P. falciparum *malaria in Kampala, Uganda. The patients belonged to one of three pre-study defined groups: uncomplicated malaria (UM), severe non-fatal malaria (SM-s), and fatal malaria (SM-f). Subset analysis was done on those with cerebral malaria (CM) or severe malaria anaemia (SMA). Monocyte ICAM-1 was measured by flow cytometry. sICAM-1 was measured by enzyme immunoassay.

**Results:**

Both sICAM-1 and monocyte cell-surface ICAM-1 followed a log-normal distribution. Median sICAM-1 concentrations increased with greater severity-of-illness: 279 ng/mL (UM), 462 ng/mL (SM-s), and 586 ng/mL (SM-f), p < 0.0001. sICAM-1 levels were not statistically different among children with CM compared to SMA. Monocyte ICAM-1 expression was significantly higher in cases of UM compared with SM-s or SM-f (p < 0.001) and was higher among the subset of patients with CM compared with SMA, p < 0.0014. The combination of sICAM-1 and cellular ICAM-1 identified distinct categories of patients (UM with low sICAM-1 and higher monocyte ICAM-1, CM with both sICAM-1 and monocyte ICAM-1 high, and SMA with sICAM-1 high but monocyte ICAM-1 low).

**Conclusion:**

In this cohort of children with *P. falciparum *malaria, sICAM-1 levels were associated with severity-of-illness. Patients with UM had higher monocyte ICAM-1 expression consistent with a role for monocyte ICAM-1 in immune clearance during non-severe malaria. Among the subsets of patients with either SMA or CM, monocyte ICAM-1 levels were higher in CM, consistent with the role of ICAM-1 as a marker of cytoadhesion. Categories of disease in pediatric malaria may exhibit specific combinations of soluble and cellular ICAM-1 expression.

## Background

Intercellular adhesion molecule-1 (ICAM-1) is an important cell adhesion molecule involved in inflammation and immunity. It is the principal ligand for leukocyte function antigen-1 (LFA1) and directs localization of leukocytes to areas of inflammation. ICAM-1 is expressed on several tissues including endothelial cells, monocytes, and lymphocytes. A soluble form of ICAM-1 circulates in plasma. Soluble ICAM-1 is released from cell-surface ICAM-1 by proteolytic cleavage in response to inflammatory cytokines or endothelial damage. The plasma half-life of circulating soluble ICAM-1 is not known[1]. ICAM-1 is one of several cell adhesion molecules important in *Plasmodium falciparum *malaria. Red blood cells, infected with *P. falciparum*, express a parasite-derived protein (*Plasmodium falciparum *erythrocyte membrane protein-1, PfEMP-1) associated with knob-like projections on the erythrocyte surface. Specific protein domains of PfEMP-1 bind to different target molecules of the infected host including blood group A and B antigens, platelet glycoprotein IV (CD36), chondroitin sulfate, complement receptor-1, and ICAM-1.

ICAM-1 expression can have both beneficial and deleterious consequences to the infected host. Monocyte ICAM-1 participates in the immune response to *P. falciparum *infection. ICAM-1 surface expression was shown to be required for the interferon-γ response of Natural Killer cells to malaria-infected red cells[2]. Early production of interferon-γ has been shown to be protective against malaria infection in both human studies[3,4] and animal models[5]. In contrast, direct adhesion of parasitized erythrocytes to ICAM-1 on cerebral endothelial cells or co-localization of monocytes to areas of erythrocyte and platelet adhesion on cerebral endothelial cells may contribute to cerebral malaria[6-8].

Evidence for the role of cell-surface ICAM-1 expression in cerebral malaria comes from histologic studies[9]. Examination of brain tissue from patients who died with cerebral malaria has demonstrated adhesion of parasitized red cells, platelets, and leukocytes to brain endothelium in association with increased endothelial expression of ICAM-1[6-8,10-12]. When dermal microvasculature, rather than cerebral microvasculature was examined, Turner *et al *observed that endothelial ICAM-1 staining did not correlate with the severity of malaria[13]. Thus, it is possible that expression of ICAM-1 on dermal endothelial cells may not reflect ICAM-1 expression on cerebral vasculature.

Laboratory studies also support a role for adhesion of parasitized erythrocytes to ICAM-1 in malaria pathogenesis. Newbold *et al *found that in-vitro binding of parasitized red cells to ICAM-1 was more common among patients with cerebral malaria compared with controls[14]. Tripathi *et al *demonstrated in-vitro that exposure of human brain microvascular endothelial cell cultures to either parasitized red cells or the supernatant of cultured parasitized red cells resulted in increased expression of ICAM-1 on brain microvascular endothelial cells, and suggested that ICAM-1 expression was driven by *P. falciparum *proteins rather than host cytokines[15]. Favre *et al *infected wild type and ICAM-1 knock out mice with *Plasmodium berghei *and found that despite similar levels of parasitaemia, wild type mice had decreased survival, greater accumulation of macrophages in brain venules and greater sequestration of parasitized red cells in alveolar capillaries[16].

Soluble plasma levels of ICAM-1 have been measured in several studies of *P. falciparum *malaria. Initial small studies identified increased levels of soluble ICAM-1 in patients with severe malaria[17-21]. In a larger case-control study conducted in the Gambia, McGuire *et al *measured soluble ICAM-1 levels in 157 children with cerebral malaria, 89 with acute non-cerebral malaria, and 156 non-malarial controls[22]. Soluble ICAM-1 levels were significantly higher (p < 0.001) among those with malaria (735 ng/mL) compared with controls (440 ng/mL), but did not discriminate those with cerebral malaria. sICAM levels also correlated with disease severity in the large study reported by Turner *et al *[13]. More recently, Tchinda *et al *reported plasma levels of soluble ICAM-1, soluble vascular adhesion molecule-1, soluble endothelial cell leukocyte adhesion molecule and TNF in children with malaria in Cameroon. Among 114 children with severe malaria, 55 children with uncomplicated malaria, and 43 non-malaria controls, soluble ICAM-1 levels were significantly higher among those with severe disease compared with either uncomplicated malaria or controls. However, among those with severe malaria syndromes, soluble ICAM-1 levels did not distinguish cerebral malaria or severe malaria anaemia from other malaria syndromes[23]. Taken together, the published literature suggests that plasma ICAM-1 concentrations are higher in patients with more severe disease, but that levels cannot be used to distinguish children with cerebral malaria.

Few clinical studies have examined ICAM-1 expression on circulating monocytes among subjects with malaria. Using flow cytometry, Jenkins *et al *examined monocyte ICAM-1 expression among 117 children with severe malaria and 239 with uncomplicated malaria[24]. Among those with both severe and non-severe malaria, they observed higher monocyte ICAM-1 expression at onset of disease compared with convalescence. Monocyte ICAM-1 fluorescence was not different among those with cerebral malaria versus those without cerebral malaria.

To further explore the relationship between cell-surface and soluble ICAM-1 among patients with severe malaria syndromes, both peripheral blood monocyte ICAM-1 expression and plasma soluble ICAM-1 concentrations were measured in a cohort of children with acute *P. falciparum *infection in Kampala, Uganda. To better understand both potentially beneficial and detrimental effects of ICAM-1 in malaria, soluble and cellular ICAM-1 measurements were obtained at presentation of illness among children with different categories of disease severity including those with uncomplicated malaria, severe malaria anaemia, and cerebral malaria.

## Methods

Patients were selected for testing from those enrolled in a larger observational case-control clinical study (enrollment dates Oct 2007-Oct 2009) of children aged 6 months to 12 years with *P. falciparum *malaria treated at Mulago Hospital in Kampala, Uganda. Prior to any testing, patients were categorized into three groups: uncomplicated malaria under treatment as outpatients (UM), severe malaria survivors treated as inpatients (SM-s), and fatal severe malaria treated as inpatients (SM-f). In addition, patients were sub-categorized according to WHO classifications of malaria syndromes as either cerebral malaria (CM) or severe malaria anaemia (SMA). Patient samples from the above 3 categories were selected from a larger study sample archive based on availability of an adequate volume of previously unthawed plasma. All patients had confirmed *P. falciparum *infection based on independent review of anonymized and clinically blinded peripheral blood smears by two experts at a university malaria confirmatory laboratory (Makerere-UCSF Molecular Laboratory, Dr Samuel Nsobya, Director). The number of parasitized red cells was determined relative to leukocyte counts. No threshold level of parasitaemia was required and assessment of quantitative parasitaemia by independent reviewers agreed within < 2%. The median concentration of parasitized red cells was 39,200/μL (range 64/μL-2,033,920/μL). Peripheral blood samples were obtained from each patient on one occasion at the time of presentation to hospital for care. Citrated plasma was separated from whole blood by centrifugation, transferred to screw-cap cryovials, and promptly frozen at -20°C shortly after phlebotomy for later testing of soluble ICAM-1 concentration. At the same phlebotomy, a peripheral whole blood sample collected in EDTA was tested for ICAM-1 expression on monocytes by flow cytometry (see below). Patients were treated in the Acute Care Unit of Mulago Hospital (a university teaching hospital setting) by Study Clinicians skilled in the management of paediatric malaria. Anti-malarial treatment was administered according to Ugandan national guidelines[25]. Parents of enrolled children gave written informed consent to participate in the study and to test stored samples. The study was approved by the Research Ethics Board of the University of Toronto, Makerere University College of Health Sciences, and the Uganda National Council for Science and Technology.

### Soluble ICAM-1 levels

Soluble ICAM-1 levels were measured using a human ELISA (DuoSet™) from R&D Systems (Minneapolis, MN). Patient plasma samples were diluted 1:1,000 with phosphate buffered saline (PBS) supplemented with 1% w/v bovine serum albumin (BSA). The ELISA was performed in duplicate according to the manufacturer's instructions with the following changes: the assay was performed in a volume of 50 μL/well; the recommended top standard was quadrupled to give a 10-point calibration curve; plasma samples were incubated overnight at 4°C prior to washing; and the final reactions were developed using Extravidin™ (Sigma-Aldrich, St Louis, MO) diluted 1:1000 in PBS with 1% BSA using a 45 min incubation followed by addition of 4-nitrophenyl phosphate substrate (Sigma-Aldrich). Optical density readings were acquired at 405 nm, and background (570 nm) was subtracted. For any given quantity (ng) of soluble ICAM-1 in circulating blood, anemic individuals will distribute that amount over a larger volume of plasma compared with non-anemic individuals, resulting in a lower measured plasma concentration. To correct for the effects of anaemia, the measured plasma concentration of ICAM-1 was converted to the concentration expected in whole blood, as follows:

Blood ICAM−1 (ng/mL blood)=plasma ICAM−1 (ng/ml plasma)×(1−Hct)

The values for blood ICAM-1 and plasma ICAM-1 are listed in Additional file 1.

### Monocyte ICAM-1 expression

ICAM-1 expression on monocytes was measured by flow cytometry using a FACSCalibur™ (BD Biosciences; San Jose, CA) with CellQuest™ (BD Biosciences, version 3.3) acquisition software and Flowjo™ (Tree Star, Ashland, OR) for analysis. Instrument setup and calibration were performed daily with CaliBRITE™ beads according to the manufacturer's recommendations. Peripheral blood, collected in EDTA, was tested within 48 hours of collection. Previous work demonstrated that monocyte ICAM-1 cell-surface levels were stable for 72 hours after sample collection and were unaffected by the platelet concentration in the sample[26]. Prior to beginning the study, serial testing of samples stored as anticoagulated whole blood in EDTA without continuous mixing at local temperatures demonstrated no statistical difference between monocyte ICAM-1 measurements at day 0 (day of phlebotomy) and day 3 of storage. During the study, 61% of patients were tested on the same day as phlebotomy or the next day, 21% on day 2, and 14% on day 3. Thus > 95% of samples were tested within three days of collection. There was furthermore no difference in monocyte ICAM-1 expression distributions as a function of the days of storage before cytometry testing (p = 0.245, ANOVA). Whole blood (100 μL) was incubated for 15 minutes at room temperature with the following monoclonal antibodies obtained from BD Biosciences: anti-CD61-FITC (clone RUU-PL7F12); anti-CD54-APC (clone HA58); and anti-CD14-PerCP (clone MΦ-P9). Following incubation, the cell suspension was lysed, washed, fixed and acquired within 24 hours. Gating strategy was based on forward scatter vs side scatter vs CD14 and CD14 vs CD61. Monocyte-platelet aggregates, identified as events double-positive for both CD14 and CD61, were eliminated from the final analysis. CD54 expression on the resulting monocyte singlets (CD14+ CD61-) was displayed as a histogram of at least 4,000 CD14 positive events and the geometric mean fluorescence intensity (MFI) channel recorded for each sample.

### Data analysis

Cumulative probability plots were used to characterize the distribution of plasma soluble ICAM-1, whole blood sICAM, and monocyte MFI. Comparison between multiple groups was done by the Kruskal-Wallis test. Comparison between two groups was done using the Wilcoxon sum test. Correlations were tested for best fit using an automated curve fitting programme [27].

## Results

Patient samples were collected between October 2007 and October 2009. Patients selected for ICAM-1 analysis were deliberately selected to represent three categories of severity-of-illness: uncomplicated malaria treated as an outpatient (UM); malaria survivors with severe malaria anaemia or cerebral malaria treated as in-patients (SM-s); and fatal malaria cases (SM-f). Clinical characteristics of the patients are shown in Table 1. All patients had confirmed *P. falciparum *infection.

**Table 1:** Clinical characteristics of the patients.

	All patients n = 160	Uncomplicated malaria **out-patients (UM)**, n = 53	Severe malaria survivors (SM-s) n = 80	Fatal malaria (SM-f),* n = 27
Age (years) median (IQR)	2.2 (1.2 - 4.4)	4.4 (2.2 - 8)	1.6 (1 - 3.1)	1.9 (1.2-3.4)

Sex (female:male)	79:81	24:29	37:43	18:9

# of days ill prior to presentation median (IQR)	3 (3-4)	3 (2-4)	3 (3-4)	4 (2-6.5)

Haemoglobin (g/dL) median (IQR)	6.3 (4.2-9.6)	10.1 (9.4 - 11.3)	4.4 (3.4 - 5.6)	5.9 (4.6 - 8.5)

WBC/μL median (IQR)	10,200 (6,800 - 14,300)	7,500 (5,200 - 9,000)	12,150 (9,375 - 16,000)	13,000 (8,500 - 25,125)

Monocytes/μL median (IQR)	66 (39 - 114)	48 (33 - 63)	94 (50 - 137)	83 (51 - 125)

Parasitized red cells/μL median (IQR)	39,600 (11,405-150,830)	37,680 (18,140-115,000)	37,010 (7,500-151,095)	118,920 (24,000-271,350)

# with cerebral malaria	39	0	24	15

# with severe malaria anaemia	54	0	47	7

# with both cerebral malaria and severe malaria anaemia	11	0	9	2

Patients with cerebral malaria or severe malaria anaemia are a subset of those with fatal or severe disease.

* Three patients with fatal malaria did not meet criteria for cerebral malaria or severe malarial anaemia.

### Soluble and cellular ICAM-1, and severity of disease

**Figure 1:**
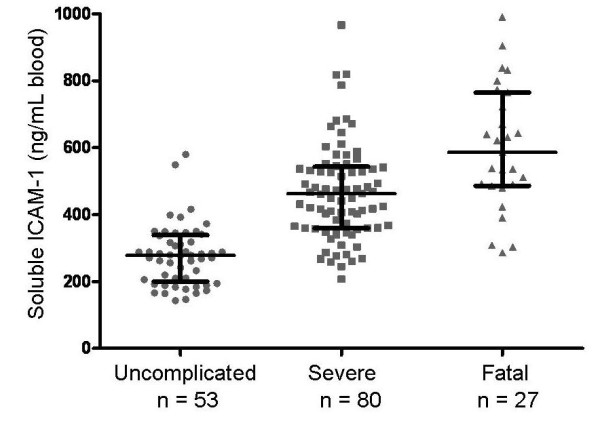
**Blood levels of soluble ICAM-1 with increasing severity of malaria**. Soluble ICAM-1 concentrations were significantly different among the three groups, p < 0.0001 (Kruskal-Wallis). Levels among fatal cases were significantly higher than among survivors with severe disease, p < 0.00065 (Wilcoxon); and higher in severe disease than among those with uncomplicated malaria, p < 1.27 × 10^-12 ^(Wilcoxon). Horizontal line is the median, brackets span the 25^th ^to 75^th ^percentile.

Soluble plasma and whole blood ICAM-1 concentrations followed a log-normal distribution. Soluble ICAM-1 concentrations progressively and significantly increased with greater severity-of-illness. The median (inter-quartile ranges) of soluble whole blood ICAM-1 concentrations measured at presentation were: 279 ng/mL (206-337) among UM patients; 462 ng/mL (360-542) among SM-s patients; and 586 ng/mL (488-743) among SM-f patients, p < 0.0001, Kruskal-Wallis test. See Figure 1. Comparison of plasma ICAM-1 concentrations, rather than whole blood ICAM-1 concentrations, gave similar results: 421 ng/mL (320-472) among UM patients; 528 ng/mL (445-653) among SM-s patients; and 718 ng/mL (647-895) among SM-f patients, p < 0.0001, Kruskal-Wallis. The concentration of parasitized red cells did not correlate with levels of sICAM-1.

**Figure 2:**
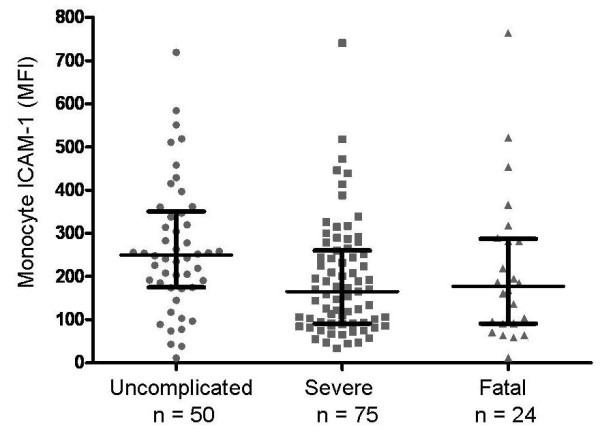
**Monocyte ICAM-1 mean fluorescence intensity (MFI) among patients with increasing severity of malaria**. Higher values were observed among subjects with uncomplicated malaria, p = 0.007 (Kruskal-Wallis). Values were not statistically different between survivors with severe disease and fatal cases, p = 0.79 (Wilcoxon). Patients with uncomplicated malaria had significantly higher values than either severe malaria survivors or fatal cases, p < 0.0017 (Wilcoxon). Horizontal line is the median, brackets span the 25^th ^to 75^th ^percentile. The number of patients in each category differs from Figure 1 because some patients with sICAM-1 measures did not have monocyte ICAM-1 measurement.

The distribution of monocyte surface ICAM-1 expression (monocyte MFI) among the three categories of severity-of-illness is shown in Figure 2. Monocyte ICAM-1 expression also followed a log-normal distribution. Median (inter-quartile range) of monocyte ICAM-1 expression among UM patients was 250 (177-345); among SM-s patients was 165 (91-256); and among SM-f patients was 177 (91-283). UM patients had significantly higher monocyte ICAM levels compared with severe or fatal cases, p = 0.007, Kruskal-Wallis test.

### Cerebral malaria versus severe malaria anaemia

**Figure 3:**
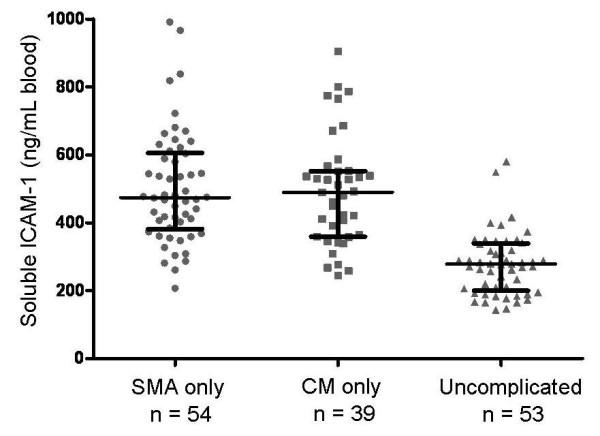
**Soluble ICAM-1 in severe malaria anaemia (SMA), cerebral malaria (CM), and uncomplicated malaria (UM)**. Concentrations were significantly different in three-way comparison, p < 0.0001 (Kruskal-Wallis). Values were not significantly different between cerebral malaria and severe malaria anaemia, p = 0.776 (Wilcoxon). Values among subjects with UM were significantly lower compared to those with SMA (p < 2.9 × 10^-12^) or compared to those with CM (p < 1.83 × 10^-9^, Wilcoxon). Horizontal line is the median, brackets span the 25^th ^to 75^th ^percentile.

To compare ICAM-1 levels among children with two major severe malaria syndromes, survivors and non-survivors were grouped into those with cerebral malaria only (CM) and those with severe malaria anaemia only (SMA). Patients with a combination of both CM and SMA (n = 11, Table 1) were not included in the comparison. Blood soluble ICAM-1 concentrations were not significantly different between children with CM (490 ng/mL; IQR 362-552) compared to those with SMA (474 ng/mL; IQR 389-600). See Figure 3. A comparison of plasma ICAM-1 concentrations (rather than blood ICAM-1 concentrations) was also not significantly different between those with CM (645 ng/mL; IQR 478-741) and those with SMA (528 ng/mL; IQR 448-668). However, levels in each severe syndrome were significantly higher than in UM patients, p < 0.0001, Kruskal-Wallis test. See Figure 3.

**Figure 4:**
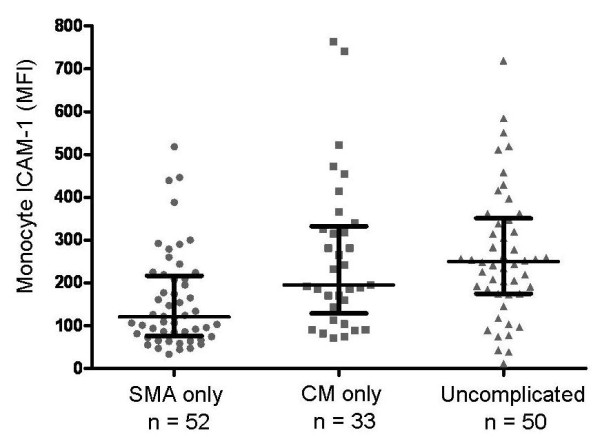
**Monocyte ICAM-1 mean fluorescence intensity (MFI) in severe malaria anaemia (SMA), cerebral malaria (CM) and uncomplicated malaria (UM)**. Monocyte ICAM-1 values were significantly different among the three groups, p < 0.0001 (Kruskal-Wallis). Values in patients with cerebral malaria were significantly higher than those with severe malaria anaemia, p < 0.0014 (Wilcoxon). Values in patients with cerebral malaria were not significantly different from levels observed in patients with uncomplicated malaria, p = 0.49. Horizontal line is the median, brackets span the 25^th ^to 75^th ^percentile. The number of patients in each category differs from Figure 3 because some patients with sICAM-1 measures did not have monocyte ICAM-1 measurement.

In contrast, monocyte surface ICAM-1 expression differed among those with CM alone compared with SMA alone. The results, shown in Figure 4, demonstrate significantly lower monocyte ICAM-1 mean fluorescence intensity among those with SMA (median MFI = 120; IQR 80-212) compared with either CM (median MFI = 195; IQR 144-326) or UM (median MFI = 250; IQR 177-345), p < 0.0001, Kruskal-Wallis test.

**Figure 5:**
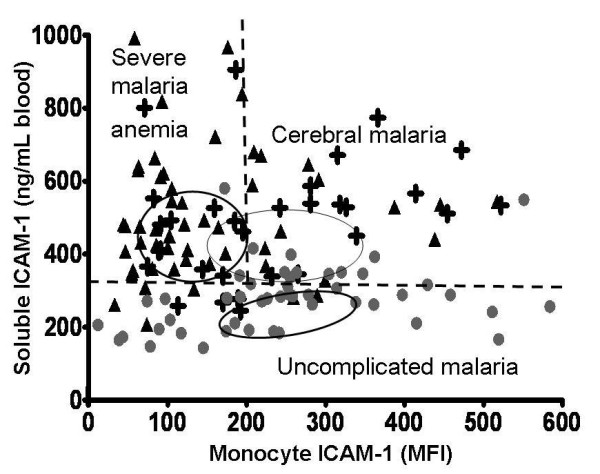
**Lack of correlation between blood levels of soluble ICAM-1 (y-axis) and monocyte cell-surface ICAM-1 (x-axis) in children with malaria**. Filled circles represent patients with uncomplicated malaria (n = 50), crosses represent patients with cerebral malaria (n = 33), and triangles represent patients with severe malarial anaemia (n = 52). Ovals represent interquartile ranges of data for each group in each dimension. Overall there is little correlation between soluble and cell-surface values. However, values cluster according to patient groups. Patients with either cerebral malaria or severe malaria anaemia have higher soluble ICAM-1 levels compared with uncomplicated malaria. Monocyte ICAM-1 levels are higher in cerebral malaria patients compared with severe malaria anaemia patients.

The relationship between soluble and monocyte ICAM-1 levels and patient categories is shown in Figure 5. For the overall dataset, no direct linear correlation between soluble ICAM-1 levels and monocyte ICAM-1 expression was found, r^2 ^= 0.02. Correlation remained weak even when values were fitted to non-linear models or were log-transformed. Among patients with UM, there was a weak positive correlation between soluble and monocyte ICAM-1 levels, slope = 0.57, r^2 ^= 0.27, p < 0.0001. Values for patients with CM, SMA, and UM clustered around different medians.

## Discussion

ICAM-1 is a major cytoadhesion ligand for PfEMP-1, which is expressed by parasitized erythrocytes. Previous histologic studies in cerebral malaria have demonstrated that ICAM-1 expression may directly contribute to cytoadhesion of malaria infected red cells or may serve to recruit leukocytes to sites of endothelial activation[6,9-12]. Studies measuring soluble ICAM-1 alone have found increased levels with greater disease severity, but no association with specific cytoadhesive syndromes[13,17,18,20-23]. Studies of ICAM-1 expression on circulating monocytes found higher levels in acute illness compared with convalescence[24]. Previous studies have not measured both soluble ICAM-1 and peripheral blood monocyte ICAM-1 expression in the same cohort of patients. This study examined the association between measurements of both soluble and cellular ICAM-1 with categories of clinical severity and with specific malaria syndromes in 160 children with documented *P. falciparum *infection.

Soluble ICAM-1 has been suggested by other authors to result principally from proteolytic cleavage (shedding) of membrane ICAM-1 expressed on endothelial cells and monocytes[28]. In this study, plasma and whole blood levels of soluble ICAM-1 followed a log-normal distribution in children with malaria. Consistent with previously published work [13,23], soluble ICAM-1 levels were found to be significantly higher among children with severe malaria syndromes or fatal malaria compared to those with uncomplicated malaria. These higher soluble ICAM-1 concentrations may reflect a combination of greater inflammatory response, increased endothelial cell activation with ICAM-1 shedding, or reduced soluble ICAM-1 clearance. Also consistent with previous work[22,23], soluble ICAM-1 levels did not distinguish patients with cerebral malaria from those with severe malaria anaemia. This was true even when plasma soluble ICAM-1 levels were corrected for the degree of anaemia in each patient.

Increased ICAM-1 expression on monocytes may be beneficial for the host with uncomplicated *P. falciparum *infection. For example, ICAM-1 expression on monocytes is required for interferon-γ release by NK cells in response to parasitized red cells[2]. In this study children with uncomplicated malaria, treated as outpatients, had significantly higher monocyte ICAM-1 expression than those with severe or fatal disease. Whether the higher observed monocyte ICAM-1 levels among outpatients were the result of higher baseline expression, increased activation in response to inflammatory cytokines, or reduced shedding due to a milder clinical course could not be determined.

In contrast, high expression of ICAM-1 on cerebral endothelial cells may contribute to cytoadhesion of parasitized red cells and cerebral malaria[11,12]. In the subset of patients with severe disease, inflammatory cytokines result in increased expression of cell-surface ICAM-1. In contrast to those with uncomplicated infection, ICAM-1 expression measured on circulating monocytes in patients with severe inflammatory malaria syndromes may reflect levels found on endothelial cells and the subset of patients with severe a cytoadhesive syndrome such as cerebral malaria might be expected to have higher levels of ICAM-1 expression compared with those with severe malaria anaemia. In addition, among such patients with severe disease, those with monocytes exhibiting high ICAM-1 expression may co-localize monocytes to parasitized red cells adherent to brain endothelium contributing further to the lesion of cerebral malaria, while in contrast, patients with low monocyte ICAM-1 may have less co-localization and reduced risk of cerebral malaria[12]. Consistent with either of these hypotheses, this study observed that patients with cerebral malaria had significantly higher monocyte ICAM-1 levels compared with those with severe malaria anaemia alone.

This study had several limitations. As an observational study, only associations may be drawn between ICAM-1 measurements and clinical outcomes. Such associations may be confounded by factors not included in the analysis. Secondly, although the study subjects were from the same region (urban Kampala, Uganda) and studied over a limited time frame (24 months), it is possible that different strains of parasites with different adhesive properties to ICAM-1 were distributed unequally among the different clinical groups. ICAM-1 was only measured at clinical presentation and it is not known if serial measurements would lead to different conclusions. Because monocytes are readily accessible, they are an appealing surrogate for endothelial cell expression. However, both regulation of ICAM-1 expression and membrane shedding from proteolytic cleavage may differ for endothelial cells and monocytes. Because the endothelium represents the largest organ in the body, direct assessment of endothelial ICAM-1 in specific organ beds affected by malaria may be a more important but more challenging source to study. Finally, soluble and monocyte ICAM-1 levels in patients with asymptomatic parasitaemia or healthy controls were not measured nor was testing done in individuals with genetic variants of ICAM-1[29-31].

Improved understanding of the role of ICAM-1 in the molecular pathology of malaria remains important. In the future, proteomics technology may allow investigators to distinguish between ICAM-1 shed from the vascular endothelium versus that shed from monocytes[32,33]. The former may provide the least invasive surrogate of endothelial expression relevant in cytoadhesion, while the latter may reveal the proportion engaged in an immune response. Further examination of cases stratified by severity and by syndrome may indeed clarify the predictive dualities of the physiology of ICAM-1 in malaria. In the meantime, to clarify further the role of ICAM-1 in malaria, measurement of both cell-surface and soluble ICAM-1 are recommended for clinical and laboratory studies of ICAM-1 and malaria.

## Competing interests

The authors declare that they have no competing interests.

## Authors' contributions

CMC, WHD, and KK conceived the study, participated in its design and coordination and helped draft the manuscript. WHD performed the statistical analysis and drafted the manuscript. IS performed the flow cytometry and LE carried out the immunoassays and helped draft the manuscript. AD and CM provided clinical care of the research subjects, collected the clinical data, and created the plasma archive. All authors contributed to and approved the final manuscript.

## Supplementary Material

Additional file 1 **Blood ICAM1 (ng/mL) and Plasma ICAM1 (ng/mL) in 160 children with P falciparum malaria**. Plasma and blood concentrations of soluble ICAM-1. Blood levels were derived from measured plasma levels and the measured haematocrit. Click here for file
