# Supplementary material for: Combined measurement of soluble and cellular ICAM-1 among children with Plasmodium falciparum malaria in Uganda
Source: Malar J. 2010 Aug 16;9:233. doi: 10.1186/1475-2875-9-233 (PMC2929237; doi:10.1186/1475-2875-9-233)
Supplement: Additional file 1 — Blood ICAM1 (ng/mL) and Plasma ICAM1 (ng/mL) in 160 children with P falciparum malaria. Plasma and blood concentrations of soluble ICAM-1. Blood levels were derived from measured plasma levels and the measured haematocrit. [file 1475-2875-9-233-S1.DOC]

**Blood ICAM1 (ng/mL) and Plasma ICAM1 (ng/mL) in 160 children with *P falciparum* malaria.**

Blood Plasma Blood Plasma Blood Plasma Blood Plasma Blood Plasma

| 451.38 | 571.4 |
| --- | --- |
| 382.29 | 455.7 |
| 347.62 | 484.8 |
| 553.54 | 655.9 |
| 429.44 | 640.0 |
| 314.65 | 429.3 |
| 329.73 | 411.6 |
| 511.63 | 613.5 |
| 242.11 | 314.8 |
| 594.12 | 655.0 |
| 186.14 | 303.7 |
| 381.72 | 506.3 |
| 373.05 | 445.2 |
| 339.16 | 406.7 |
| 384.75 | 512.3 |
| 361.17 | 432.5 |
| 284.75 | 423.7 |
| 147.80 | 231.3 |
| 253.23 | 358.7 |
| 531.68 | 766.1 |
| 164.86 | 241.0 |
| 185.22 | 211.7 |
| 298.40 | 353.1 |
| 318.26 | 419.3 |
| 446.05 | 521.7 |
| 562.26 | 771.3 |
| 463.40 | 533.3 |
| 317.31 | 358.1 |
| 258.65 | 359.2 |
| 310.69 | 384.0 |
| 501.76 | 602.4 |
| 325.06 | 371.9 |
| 169.61 | 252.4 |
| 274.66 | 305.9 |
| 273.51 | 301.6 |
| 416.97 | 477.1 |
| 274.49 | 383.9 |
| 291.96 | 414.7 |
| 135.45 | 210.3 |
| 527.58 | 613.5 |
| 265.46 | 379.8 |
| 209.50 | 305.0 |
| 468.86 | 632.7 |
| 540.16 | 689.0 |
| 346.99 | 485.3 |
| 155.86 | 254.7 |
| 407.58 | 492.2 |
| 523.78 | 614.8 |
| 167.88 | 250.6 |
| 757.83 | 924.2 |
| 403.09 | 470.9 |
| 467.47 | 530.0 |
| 185.34 | 312.5 |
| 515.08 | 667.2 |
| 457.90 | 503.2 |
| 531.10 | 583.0 |
| 618.64 | 703.8 |
| 247.19 | 366.8 |
| 248.23 | 279.5 |
| 352.70 | 470.9 |
| 477.36 | 612.0 |
| 267.50 | 396.9 |
| 369.10 | 414.7 |
| 260.52 | 419.5 |
| 591.85 | 702.9 |
| 337.79 | 455.9 |
| 798.39 | 1101.2 |
| 324.37 | 436.0 |
| 268.04 | 432.3 |
| 561.43 | 633.0 |
| 325.53 | 512.6 |
| 1410.95 | 2316.8 |
| 619.62 | 727.3 |
| 451.17 | 497.4 |
| 387.50 | 440.3 |
| 319.36 | 479.5 |
| 895.83 | 1083.2 |
| 156.96 | 235.7 |
| 817.21 | 971.7 |
| 267.21 | 412.4 |
| 262.67 | 360.8 |
| 758.79 | 941.4 |
| 982.81 | 1113.0 |
| 326.57 | 479.5 |
| 402.65 | 611.9 |
| 570.10 | 832.3 |
| 192.24 | 290.8 |
| 820.75 | 929.5 |
| 241.15 | 345.5 |
| 509.55 | 613.9 |
| 447.21 | 505.3 |
| 268.59 | 419.0 |
| 500.41 | 571.2 |
| 475.19 | 743.7 |
| 394.17 | 585.7 |
| 551.63 | 619.8 |
| 510.75 | 624.4 |
| 249.01 | 346.3 |
| 488.16 | 652.6 |
| 256.69 | 296.4 |
| 781.30 | 1143.9 |
| 241.59 | 378.1 |
| 625.13 | 683.9 |
| 305.00 | 426.0 |
| 350.32 | 506.24 |
| 425.38 | 489.51 |
| 966.31 | 1121.01 |
| 403.36 | 471.77 |
| 146.75 | 218.05 |
| 268.85 | 329.07 |
| 467.29 | 525.64 |
| 256.15 | 378.92 |
| 818.92 | 920.13 |
| 183.28 | 291.39 |
| 188.17 | 282.11 |
| 639.75 | 703.02 |
| 611.19 | 668.7 |
| 463.13 | 495.86 |
| 360.80 | 402.68 |
| 268.20 | 421.04 |
| 468.73 | 516.79 |
| 241.54 | 449.79 |
| 566.01 | 710.18 |
| 308.08 | 343.46 |
| 373.45 | 418.2 |
| 359.22 | 420.14 |
| 818.48 | 908.41 |
| 491.47 | 578.2 |
| 407.02 | 468.38 |
| 245.03 | 350.54 |
| 316.13 | 458.82 |
| 531.57 | 605.43 |
| 398.45 | 569.22 |
| 669.57 | 758.29 |
| 722.00 | 853.43 |
| 546.09 | 639.45 |
| 191.88 | 303.13 |
| 310.56 | 460.77 |
| 232.65 | 374.63 |
| 210.14 | 347.91 |
| 166.32 | 284.3 |
| 514.36 | 596.71 |
| 486.43 | 652.05 |
| 392.36 | 619.84 |
| 533.47 | 795.04 |
| 526.76 | 674.47 |
| 552.39 | 674.47 |
| 461.71 | 584.44 |
| 306.50 | 456.1 |
| 645.18 | 772.67 |
| 680.55 | 770.72 |
| 670.86 | 825.16 |
| 440.46 | 525.61 |
| 544.24 | 657.3 |
| 685.29 | 850.24 |
| 282.56 | 430.07 |
| 318.32 | 472.29 |
| 288.18 | 464.81 |
| 279.49 | 468.94 |
| 449.51 | 525.13 |
